# Aztreonam-Avibactam Susceptibility Testing Program for Metallo-Beta-Lactamase-Producing *Enterobacterales* in the Antibiotic Resistance Laboratory Network, March 2019 to December 2020

**DOI:** 10.1128/AAC.00486-21

**Published:** 2021-07-16

**Authors:** Amelia Bhatnagar, Sandra Boyd, Sarah Sabour, Janine Bodnar, Elizabeth Nazarian, Nadine Peinovich, Christine Wagner, Bradley Craft, Paula Snippes Vagnone, Justin Simpson, Victoria N. Stone, Michelle Therrien, Allen Bateman, Danielle Lower, Jennifer Y. Huang, Stephanie Gumbis, David Lonsway, Joseph D. Lutgring, Maria Karlsson, Allison C. Brown

**Affiliations:** a Division of Healthcare Quality Promotion, Centers for Disease Control and Prevention, Atlanta, Georgia, USA; b Wadsworth Center, New York State Department of Health, Albany, New York, USA; c Minnesota Department of Health, St. Paul, Minnesota, USA; d Tennessee Department of Health, Nashville, Tennessee, USA; e Wisconsin State Laboratory of Hygiene, Madison, Wisconsin, USA

**Keywords:** *Enterobacterales*, MBL, antibiotic resistance, antimicrobial combinations, aztreonam-avibactam, beta-lactamases, carbapenemases, susceptibility testing

## Abstract

Aztreonam-avibactam is a drug combination pending phase 3 clinical trials and is suggested for treatment of severe infections caused by metallo-beta-lactamase (MBL)-producing *Enterobacterales* by combining ceftazidime-avibactam and aztreonam. Beginning in 2019, four Antibiotic Resistance Laboratory Network regional laboratories offered aztreonam-avibactam susceptibility testing by broth microdilution. For 64 clinical isolates tested, the MIC_50_ and MIC_90_ values of aztreonam-avibactam were 0.5/4 μg/ml and 8/4 μg/ml, respectively. Aztreonam-avibactam displayed potent *in vitro* activity against the MBL-producing *Enterobacterales* tested.

## INTRODUCTION

Metallo-beta-lactamase (MBL)-producing *Enterobacterales* are an emerging public health threat (1). Data collected between 2017 and 2019 by the Centers for Disease Control and Prevention’s (CDC) Antibiotic Resistance Laboratory Network (AR Lab Network) showed that MBL carbapenemase genes (*bla*_IMP_, *bla*_NDM_, and *bla*_VIM_) were present in 4.1% (1,743/42,423) of all carbapenem-resistant *Enterobacterales* (CRE) tested (2). These MBL genes, often located on mobile plasmids, commonly confer resistance to multiple beta-lactam agents, limiting the number of effective treatment options (3).

Recent case reports (4, 5) and *in vitro* studies (6–9) suggest that a novel drug combination, aztreonam-avibactam, may have efficacy against infections caused by MBL-producing *Enterobacterales*. Phase 3 clinical trials are pending, but the combination therapy of aztreonam-avibactam can currently be achieved by administering two Food and Drug Administration (FDA)-approved drugs, aztreonam and ceftazidime-avibactam. Additionally, the Infectious Diseases Society of America suggests this combination as a potential treatment option for infections caused by MBL-producing CRE (10).

In the absence of commercially available antimicrobial susceptibility tests, the CDC validated a new method for preparing broth microdilution (BMD) panels for aztreonam-avibactam antimicrobial susceptibility testing (AST) (11). Thereafter, the CDC deployed this test to four regional laboratories of the AR Lab Network to provide on-demand AST of aztreonam-avibactam for MBL-producing *Enterobacterales* (12). This study describes *in vitro* aztreonam-avibactam susceptibility of isolates tested from March 2019 to December 2020.

*Enterobacterales* isolates that met ≥1 of the following criteria were eligible for aztreonam-avibactam AST at the AR Lab Network regional laboratories: (i) PCR-positive for ≥1 MBL gene (*bla*_NDM_, *bla*_VIM_, or *bla*_IMP_) or (ii) not susceptible to all beta-lactams tested by the submitting laboratory, including at least ceftazidime-avibactam and/or meropenem-vaborbactam.

The presence of carbapenemase genes was confirmed using CDC lab-developed real-time PCR methods or the GeneXpert Carba-R assay (Cepheid, Sunnyvale, CA). AST was performed in compliance with Clinical Laboratory Improvement Amendments guidelines for aztreonam, ceftazidime-avibactam, and aztreonam-avibactam using BMD panels prepared by the D300e digital dispenser (HP, Corvallis, OR) (11). The CDC provided laboratories with drug stock aliquots of aztreonam, ceftazidime, and avibactam and tubes containing 11 ml of cation-adjusted Mueller-Hinton broth. Quality control strains used were Escherichia coli (ATCC 25922), Pseudomonas aeruginosa (ATCC 27853), and Klebsiella pneumoniae (ATCC 700603). Participating laboratories also performed AST using Sensititre GNX2F BMD panels (Thermo Fisher Scientific, Waltham, MA) validated to have a final CFU/ml of approximately 5 × 10^5^. Interpretive criteria were applied according to Clinical and Laboratory Standards Institute guidelines when available (13). Due to lack of interpretive criteria for aztreonam-avibactam, only an MIC was reported to submitters. Previous studies demonstrated that aztreonam-avibactam provided equivalent *in vitro* susceptibility for ceftazidime-avibactam plus aztreonam in highly resistant *Enterobacterales*; therefore, although patients receive ceftazidime-avibactam plus aztreonam, MICs for the triple combination were not reported (14). Results were typically reported to submitters within three working days.

Laboratories reported results to the CDC using a Research Electronic Data Capture (REDCap) database (Vanderbilt University, Nashville, TN). Results from one isolate per species per patient were included. If multiple isolates of the same species were submitted, the isolate with the earliest collection date was included. Data were analyzed using Statistical Analysis Software (v9.4; SAS Institute, Cary, NC).

Sixty-four isolates from 24 states submitted for aztreonam-avibactam AST met the inclusion criteria (Table S1). The organisms tested included Escherichia coli (*n* = 28), Klebsiella pneumoniae (*n* = 24), Enterobacter cloacae complex (*n* = 10), Morganella morganii (*n* = 1), and Proteus mirabilis (*n* = 1) (Table 1). Fifty-five isolates carried *bla*_NDM_, eight harbored *bla*_NDM_ and *bla*_OXA-48-like_, and one harbored *bla*_NDM_ and *bla*_KPC_ genes. Specimen sources were urine (*n* = 23), blood (*n* = 14), respiratory (*n* = 13), rectal swabs (*n* = 5), and other (*n* = 9).

**TABLE 1:** MIC distributions for 64 *Enterobacterales* isolates tested against ceftazidime-avibactam, aztreonam, and aztreonam-avibactam: AR Lab Network, March 2019 to December 2020

Isolate categories	Antimicrobial agent^*a*^	No. of isolates at each MIC (μg/ml) for each antimicrobial agent^*b*^													MIC_50_^*c*^	MIC_90_^*c*^
		≤0.03	0.06	0.12	0.25	0.5	1	2	4	8	16	32	64	>64		
All (64)	CZA													64	>64/4	>64/4
	ATM			2		1		1		3	4	4	8	41	>64	>64
	AZA		7	7	11	13	7	1	9	7	2				0.5/4	8/4
Escherichia coli (28)	CZA													28	>64/4	>64/4
	ATM							1		3		2	3	19	>64	>64
	AZA		1	4		3	2	1	8	7	2				4/4	8/4
NDM (27)	CZA													27	>64/4	>64/4
	ATM							1		3		2	3	18	>64	>64
	AZA		1	4		3	2	1	8	6	2				4/4	8/4
NDM & OXA-48-like (1)	CZA													1		
	ATM													1		
	AZA									1						
Klebsiella pneumoniae (24)	CZA													24	>64/4	>64/4
	ATM					1						1	5	17	>64	>64
	AZA		3	2	8	8	2		1						0.25/4	1/4
NDM (17)	CZA													17	>64/4	>64/4
	ATM					1							4	12	>64	>64
	AZA		2	2	7	4	1		1						0.25/4	1/4
NDM & OXA-48-like (7)	CZA													7		
	ATM											1	1	5		
	AZA		1		1	4	1									
Enterobacter cloacae complex, NDM (10)	CZA													10	>64/4	>64/4
	ATM			2							2	1		5	32	>64
	AZA		2	1	3	1	3								0.25/4	1/4
Morganella morganii, NDM & KPC (1)	CZA													1		
	ATM										1					
	AZA					1										
Proteus mirabilis, NDM (1)	CZA													1		
	ATM										1					
	AZA		1													

a ATM, aztreonam; AZA, aztreonam-avibactam; CZA, ceftazidime-avibactam. Avibactam is at a constant concentration of 4 μg/ml when in combination.

b Gray shading indicates the not susceptible ranges for CZA and ATM. AZA does not have interpretive criteria.

c MIC_50_ and MIC_90_ were calculated only for groups with >9 isolates.

All isolates displayed resistance to ceftazidime-avibactam, and 93.8% (60/64) exhibited not susceptible MICs for aztreonam (≥8 μg/ml). Aztreonam-avibactam MICs ranged from 0.06/4 μg/ml to 16/4 μg/ml, and the MIC_50_ and MIC_90_ were 0.5/4 μg/ml and 8/4 μg/ml, respectively. For isolates not susceptible to aztreonam and ceftazidime-avibactam (*n* = 60), combining avibactam with aztreonam reduced the MIC of aztreonam by ≥4-fold in all isolates, with a median reduction of ≥128-fold. Moreover, the addition of avibactam restored susceptibility to aztreonam (≤4 μg/ml) in 85% (51/60) of these highly resistant isolates. In aztreonam-susceptible isolates (*n* = 4), avibactam had a negligible effect (no reduction or a 2-fold reduction) in three isolates with very low aztreonam MICs (≤0.5 μg/ml). For one isolate with reduced susceptibility to aztreonam alone (2 μg/ml), the presence of avibactam resulted in a 16-fold MIC reduction. Three NDM-producing isolates (2 K. pneumoniae and 1 P. mirabilis) were resistant to aztreonam and ceftazidime-avibactam and not susceptible to all other antimicrobials on the GNX2F panel (Table S2) but remained vulnerable to aztreonam-avibactam (0.06/4 to 1/4 μg/ml).

When examining MIC distributions by organism, E. coli demonstrated higher MIC_50_ and MIC_90_ results (4/4 μg/ml and 8/4 μg/ml, respectively) than others (Table 1). A similar observation was made in a large study of 275 NDM-producing *Enterobacterales*; the MIC_50_ and MIC_90_ were higher in 115 E. coli isolates (2/4 μg/ml and 8/4 μg/ml, respectively) than the MIC_50_ and MIC_90_ observed in 125 K. pneumoniae isolates (0.25/4 μg/ml and 0.5/4 μg/ml, respectively) (15). In our study, all 9 isolates for which avibactam did not restore aztreonam susceptibility were E. coli. This phenomenon could be explained by polymorphisms in the penicillin-binding protein 3 (16, 17); additional studies are needed to confirm their presence and role in our isolates.

One limitation of our study is the sample size—a small convenience sample of 64 highly resistant NDM-producing *Enterobacterales*. While our findings may not be generalizable to other organisms or other MBL carbapenemases circulating in *Enterobacterales*, this subset of isolates represents a rich collection of very rarely detected mechanisms, and the strict inclusion criteria employed for testing are consistent with the clinical and microbiological characteristics of infections for which aztreonam-avibactam (i.e., aztreonam and ceftazidime-avibactam) is an option for therapeutic consideration. Another limitation is that no patient outcome data were collected to ascertain whether aztreonam and ceftazidime-avibactam were subsequently coadministered for treatment and whether such treatment was effective. Finally, aztreonam-avibactam clinical breakpoints have not yet been established; therefore, the MIC data reported here must be interpreted cautiously.

In summary, organisms harboring MBLs confer resistance to many available antimicrobial agents and present clinicians with few, if any, effective treatment options. While our *in vitro* data demonstrate that aztreonam-avibactam has considerable activity against *Enterobacterales* coharboring MBL carbapenemases, specifically NDM, and certain other beta-lactamases capable of hydrolyzing aztreonam, more studies are necessary to assess the *in vivo* efficacy of aztreonam-avibactam. As of 2021, all seven AR Lab Network regional laboratories now offer aztreonam-avibactam AST for MBL-producing *Enterobacterales*, filling a critical gap by providing rapid results to help inform clinical treatment decisions (12). Interested health care and public health professionals can contact their AR Lab Network regional laboratory for more information.
