# Supplementary material for: Aztreonam-Avibactam Susceptibility Testing Program for Metallo-Beta-Lactamase-Producing Enterobacterales in the Antibiotic Resistance Laboratory Network, March 2019 to December 2020
Source: Antimicrob Agents Chemother. 2021 Jul 16;65(8):e00486-21. doi: 10.1128/AAC.00486-21 (PMC8284474; doi:10.1128/AAC.00486-21)
Supplement: Supplemental file 1 — Supplemental Table S1. Download AAC00486-21_Supp_1_seq3.pdf, PDF file, 0.04 MB [file aac00486-21_supp_1_seq3.pdf]

Table S1: Geographical Distribution of 64 MBL-producing Enterobacterales Isolates Submitted for Aztreonam-Avibactam AST—Antibiotic Resistance Laboratory Network, March 2019–December 2020

State of Origin	Number Submitted
Connecticut	8
Maryland	7
New York	7
Minnesota	5
Georgia	4
Massachusetts	4
California	3
Missouri	3
Arkansas	2
Colorado	2
Kentucky	2
North Carolina	2
Nebraska	2
New Jersey	2
Wisconsin	2
Texas	1
Indiana	1
Michigan	1
Mississippi	1
Pennsylvania	1
Rhode Island	1
South Carolina	1
Virginia	1
Washington	1
